# A surgical case of posterior mediastinum chordoma requiring differentiation from neurogenic tumor

**DOI:** 10.1186/s44215-026-00241-w

**Published:** 2026-03-30

**Authors:** Morimichi Nishihira, Yoko Karube, Yusuke Shimizu, Toru Harasawa, Natsumi Ishikawa, Satoru Kobayashi

**Affiliations:** 1 Department of General Thoracic Surgery, Dokkyo Medical University Saitama Medical Center, Saitama, Japan; 2 Dokkyo Medical University, Saitama Medical Center 2-1-50 MinaMi- Koshigaya, Koshigaya, Saitama, 343-8555 Japan

**Keywords:** Chordoma, Mediastinal tumor, RATS

## Abstract

**Introduction:**

Chordoma is a malignant bone and soft tissue tumor derived from remnants of the embryonic notochord. It accounts for 1–4% of all malignant bone tumors and most commonly arises in the sacrum and skull base. Mediastinal chordomas are extremely rare, comprising only 1–3% of all chordomas.

**Case presentation:**

A 46-year-old male patient presented to the clinic for a routine check-up and was referred to our hospital after an abnormal chest shadow was detected during a routine physical examination. A contrast-enhanced computed tomography scan revealed a spindle-shaped lesion with an extrapleural sign adjacent to the vertebral body at the Th2-3 level. The lesion was predominantly cystic with no contrast enhancement. There was no evidence of surrounding invasion, bone destruction, or extension into the intervertebral foramen. We suspected a neurogenic tumor with cystic degeneration. Complete resection was considered feasible, and we performed a robot-assisted mediastinal tumor resection. The lesion was cystic, soft, and showed no apparent connection to the nerves. It was excised through total circumferential dissection, with no evident invasion into the surrounding tissue. Complete resection was successfully achieved, and the patient was discharged without complications during the postoperative course. Pathological examination confirmed the diagnosis of chordoma. Two years post-surgery, the patient remains alive without recurrence.

**Conclusion:**

We report a case of a primary posterior mediastinal chordoma that was successfully resected using robot-assisted surgery.

## Introduction

Chordoma is a rare malignant tumor that originates from notochordal cells. Mediastinal chordomas are exceedingly uncommon, comprising only 1–3% of all chordoma cases [1, 2]. Computed tomography (CT) scans typically reveal characteristic findings, including bone destruction and cyst formation. In this study, we encountered a slowly growing chordoma in the posterior mediastinum that exhibited no signs of bone destruction.

## Case presentation

**Fig. 1:**
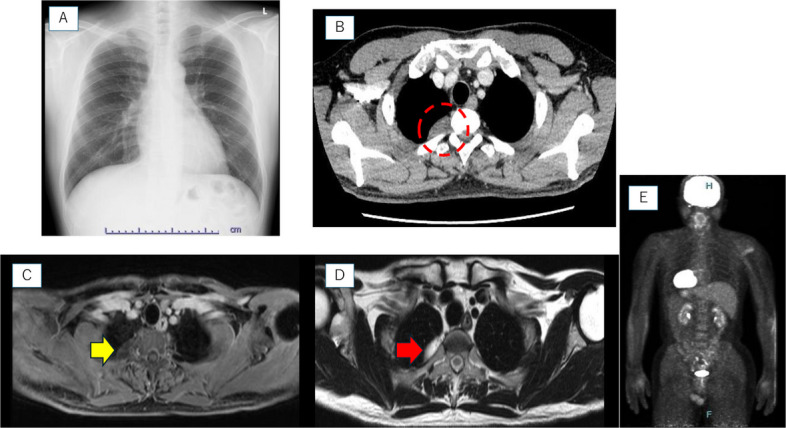
Representative images from the chest examination. **A** Chest X-ray shows no abnormal shadows. **B** The contrast-enhanced computed tomography (CT) scan reveals a spindle-shaped lesion with an extrapleural sign adjacent to the vertebral body. **C** T1-weighted magnetic resonance imaging (MRI) reveals a low-signal lesion (yellow arrow). **D** T2-weighted MRI shows a high-signal lesion (red arrow). **E** ^18^F-fluorodeoxyglucose positron emission tomography (FDG-PET)/CT imaging reveals no significant FDG accumulation

A 46-year-old male patient who was referred to our hospital after an abnormal chest shadow was detected during a routine physical examination. His routine blood tests revealed no abnormalities. A chest X-ray showed no abnormal shadow (Fig. 1A), and a contrast-enhanced CT scan revealed a spindle-shaped lesion with an extrapleural sign adjacent to the vertebral body at the Th2-3 level. The tumor measured 3.1 × 1.6 × 1.7 cm. The lesion was predominantly cystic with no contrast enhancement. No evidence of surrounding invasion, bone destruction, or extension into the intervertebral foramen was observed (Fig. 1B).

T1-weighted magnetic resonance imaging (MRI) revealed a low-signal lesion (Fig. 1C). T2-weighted MRI revealed a high-signal lesion (Fig. 1D). Similar to the CT findings, there was no evidence of invasion into the surrounding structures or bone destruction, and no obvious extension into the intervertebral foramina or spinal canal was observed. ^18^F-fluorodeoxyglucose positron emission tomography (FDG-PET)/CT showed no significant FDG uptake (Fig. 1E).

We suspected a neurogenic tumor with cystic degeneration. Complete resection was considered feasible, and we performed a robot-assisted mediastinal tumor resection.

**Fig. 2:**
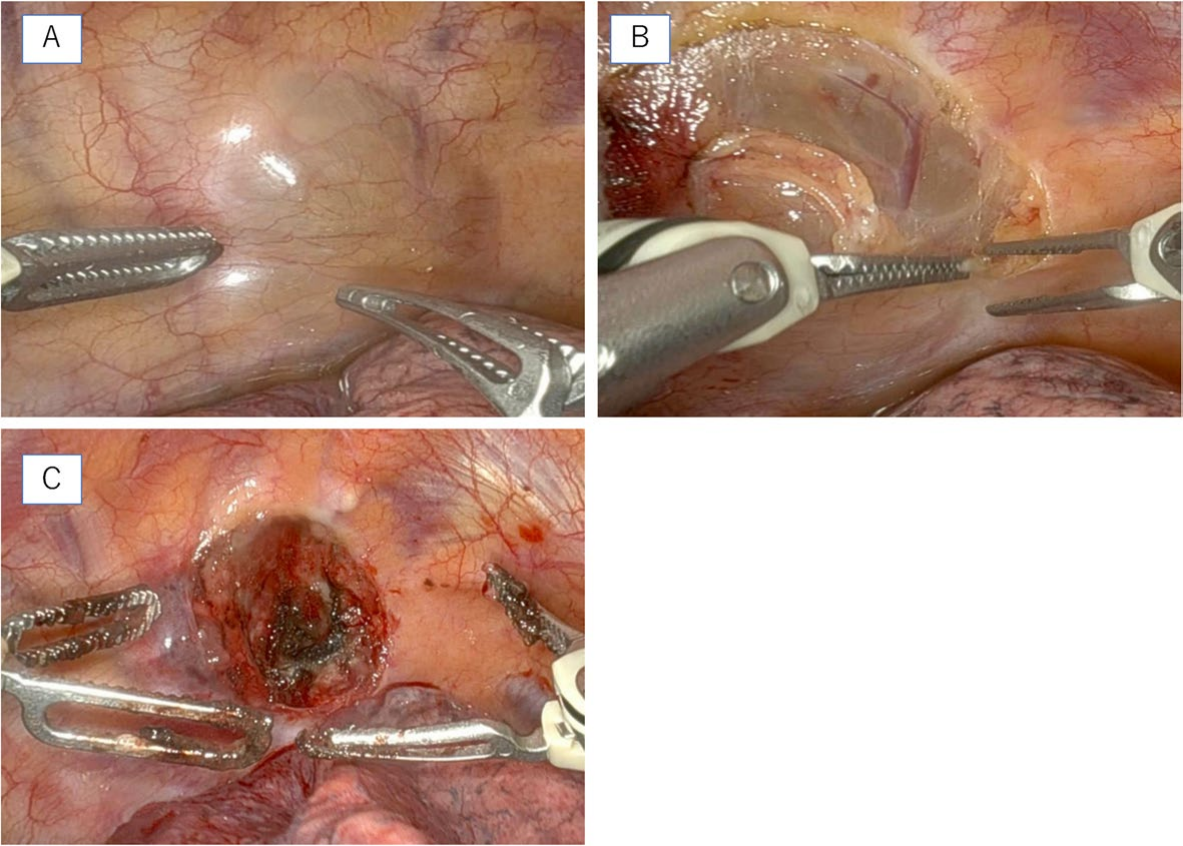
Intraoperative findings in the patient. **A** Intraoperative findings show that the tumor is soft and suspected to have cystic degeneration, consistent with the imaging findings. **B** The tumor is carefully dissected from the chest wall. **C** Intraoperative image after tumor resection

**Fig. 3:**
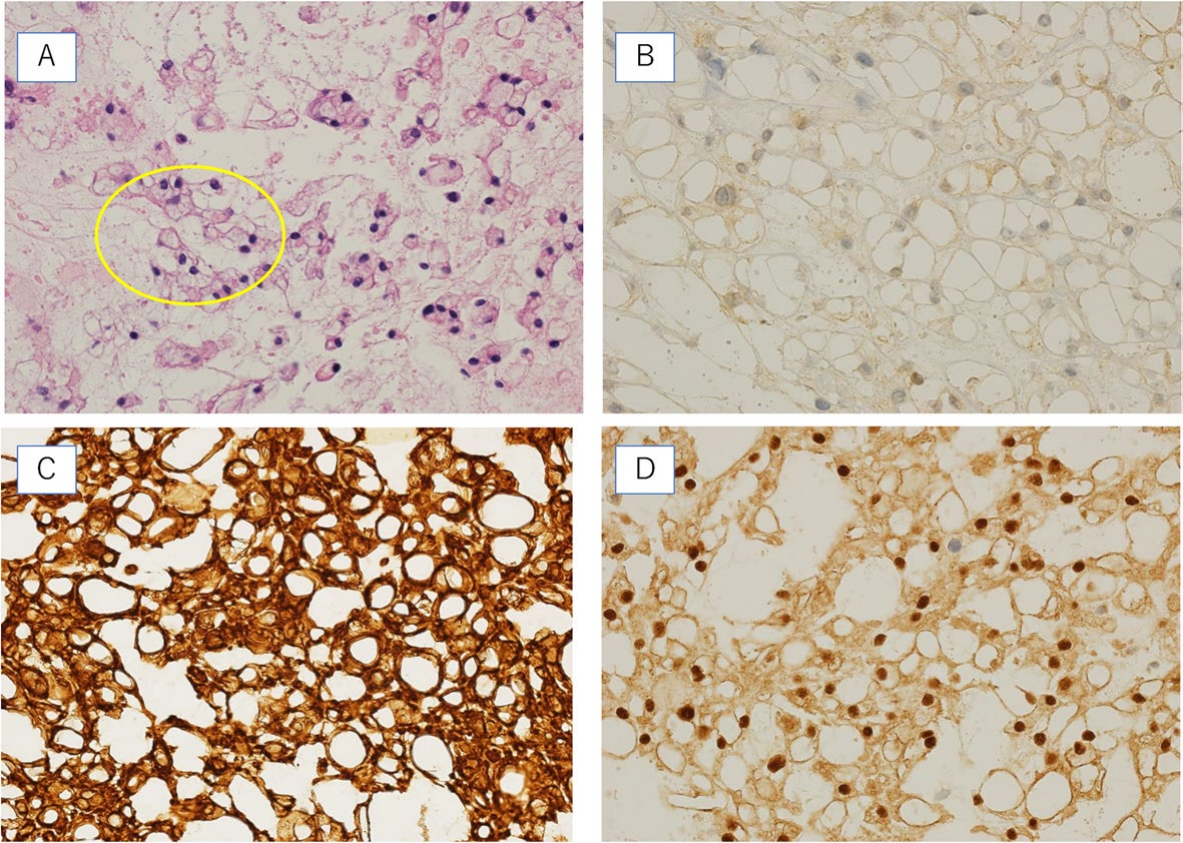
Pathological findings in the patient. **A** Hematoxylin and eosin-stained image shows the characteristic “physaliphorous cell” morphology. **B** The tumor cells show weak positive staining with antibodies against S-100. **C** The tumor cells show strong positive staining with antibodies against EMA. **D** The tumor cells shows strong positive staining with antibodies against brachyury

The lesion was cystic, soft, and showed no apparent connection to the nerves. It was excised through total circumferential dissection, with no evident invasion into the surrounding tissue (Fig. 2A, B, C). Furthermore, as the tumor was encapsulated, it was carefully dissected circumferentially without breaching the capsule and then removed. Complete resection was successfully achieved. The patient was discharged without complications during the postoperative course. Hematoxylin and eosin staining revealed the characteristic “physaliphorous cell” morphology (Fig. 3A). Immunohistochemical staining revealed that the tumor cells were positive for AE1/AE3, EMA, and Brachyury(Fig. 3B, C, D). These findings confirmed the diagnosis of chordoma.

Postoperative radiation therapy was not administered due to the patient’s refusal, and the patient is currently under observation.

Two years post-surgery, the patient remains alive without recurrence.

## Discussion

Chordoma is an extremely rare condition, with an incidence of 8 per 10 million people. Although it is considered histologically low grade, it has a high recurrence rate [3]. The 5-year survival rate is approximately 50% [4]. Imaging findings are typically characterized by partial calcification and destruction of surrounding bone tissue [5, 6]. The diagnosis is confirmed pathologically through immunoreactivity with epithelial markers such as S-100, epithelial membrane antigen (EMA), and cytokeratin. Recently, the sensitivity and specificity of Brachyury and cytokeratin staining have been reported to be very high [7]. Chordomas are considered to benefit from surgical resection with adequate margins [8, 9]. Our patient presented with a cystic lesion without bone destruction, and robot-assisted surgery enabled minimally invasive and complete resection. Notably, chordomas are typically characterized by bone destruction and compression of surrounding nerves.

Pathological examination revealed that the tumor was encapsulated with no evidence of exposure; however, since the final diagnosis was chordoma, the resection margin was considered inadequate. There have been no prior reports on the appropriate surgical margin for mediastinal chordoma. However, in sacral chordomas, it has been reported that securing a margin of at least 1.5 mm was associated with the lowest risk of local recurrence [10].

As mentioned above, no large cohort studies have been reported regarding recurrence patterns of mediastinal chordoma. Due to the anatomical relationships with surrounding organs such as the great vessels, trachea, and esophagus, achieving adequate surgical margins is often difficult, and several reports have described local recurrence in cases that resulted in R1 or R2 resections [11–13]. Additionally, cases of isolated pulmonary metastasis following initial treatment, as well as early recurrences attributed to intraoperative tumor seeding, have also been documented [14, 15].

In cases with insufficient surgical margins, the addition of radiotherapy is recommended to achieve local control. In the present case, as there was a possibility of inadequate margins, adjuvant radiotherapy was considered; however, the patient did not consent, and thus close observation was chosen. The patient has remained recurrence-free for two years postoperatively; however, since recurrence has been reported to occur from several months to several years after surgery (with a mean of approximately 40 months), careful long-term follow-up remains essential [16].

The mechanism by which chordomas cause bone destruction is not yet fully understood. However, it has been reported that chordoma cells may exhibit osteolytic activity at the tumor-bone interface, in addition to the normal bone resorption process carried out by osteoclasts. The patient had a lesion in contact with the Th2-3 vertebral level, but imaging findings showed no destruction of the thoracic spine. As a result, a neurogenic tumor was suspected, and the patient proceeded with surgery. Intraoperative findings revealed no bone destruction, and the tumor was easily resected with robotic assistance. Robotic surgery is considered beneficial due to its high-definition capabilities and provides a close field of view, aiding in the assessment of tumor invasion. A previous report described a chordoma arising in the posterior mediastinum without bone destruction, which was successfully removed thoracoscopically. The operation was performed without complications, similar to the present case [17, 18]. Chordomas often extend into surrounding tissues and bone, necessitating joint surgery with orthopedic intervention for complete resection of the lesion. Minimally invasive surgeries, such as VATS and RATS, are considered useful for chordomas without bone destruction, as demonstrated in this case.

## Conclusion

We report a case of a chordoma arising in the posterior mediastinum without bone destruction that was completely resected using minimally invasive robotic-assisted surgery.

## Data Availability

Not applicable.

## Abbreviations

CT: Computed tomography
MRI: Magnetic resonance imaging
FDG-PET: ^18^F-fluorodeoxyglucose positron emission tomography
